# Risk Factors and Cardiovascular Disease in the Elderly

**DOI:** 10.31083/j.rcm2306188

**Published:** 2022-05-25

**Authors:** Pablo Díez-Villanueva, César Jiménez-Méndez, Clara Bonanad, Sergio García-Blas, Ángel Pérez-Rivera, Gonzalo Allo, Héctor García-Pardo, Francesc Formiga, Miguel Camafort, Manuel Martínez-Sellés, Albert Ariza-Solé, Ana Ayesta

**Affiliations:** ^1^Cardiology Department, Hospital Universitario de La Princesa, 28006 Madrid, Spain; ^2^Cardiology Department, Hospital Universitario Puerta del Mar, 11009 Cádiz, Spain; ^3^Cardiology Department, Hospital Clínico Universitario de Valencia, 46010 Valencia, Spain; ^4^Cardiology Department, Hospital Clínico Universitario de Burgos, 09006 Burgos, Spain; ^5^Universidad Isabel I, 09003 Burgos, Spain; ^6^Endocrinology Department, Hospital Universitario Doce de Octubre, 28041 Madrid, Spain; ^7^Cardiology Department, Hospital Universitario Rio Hortega, 47012 Valladolid, Spain; ^8^Internal Medicine Department, Hospital Universitario de Bellvitge, L'Hospitalet de Llobregat, IDIBELL, 08907 Barcelona, Spain; ^9^Internal Medicine Department, Hospital Clinic, 08036 Barcelona, Spain; ^10^Cardiology Department, Hospital General Universitario Gregorio Marañón, CIBERCV, 28007 Madrid, Spain; ^11^School of Medicine, Universidad Complutense de Madrid, 28040 Madrid, Spain; ^12^School of Biomedical Sciences, Universidad Europea de Madrid, 28670 Madrid, Spain; ^13^Cardiology Department, Hospital Universitario de Bellvitge, L'Hospitalet de Llobregat, IDIBELL, 08907 Barcelona, Spain; ^14^Cardiology Department, Hospital Central de Asturias, 33011 Oviedo, Spain

**Keywords:** cardiovascular risk factors, cardiovascular disease, elderly

## Abstract

Age is associated with increased cardiovascular risk factors and cardiovascular disease, which constitutes the leading cause of morbidity and mortality in elderly population. In this text we thoroughly review current evidence regarding the impact on cardiovascular disease of the most important cardiovascular risk factors, especially prevalent and common in the elderly population. Diagnosis and treatment approaches are also addressed, also highlighting the importance of adequate primary and secondary prevention and management. Also, the relationship between cardiovascular disease and some comorbidities and geriatric conditions, such as frailty, particularly common in the elderly, is reviewed, together with some other issues, less often addressed but closely related to ageing, such as genetics, structural and electrical heart changes and oxidative stress. All such questions are of great importance in the comprehensive approach of risk factors and cardiovascular disease in the elderly.

## 1. Introduction

**Fig. 1.**
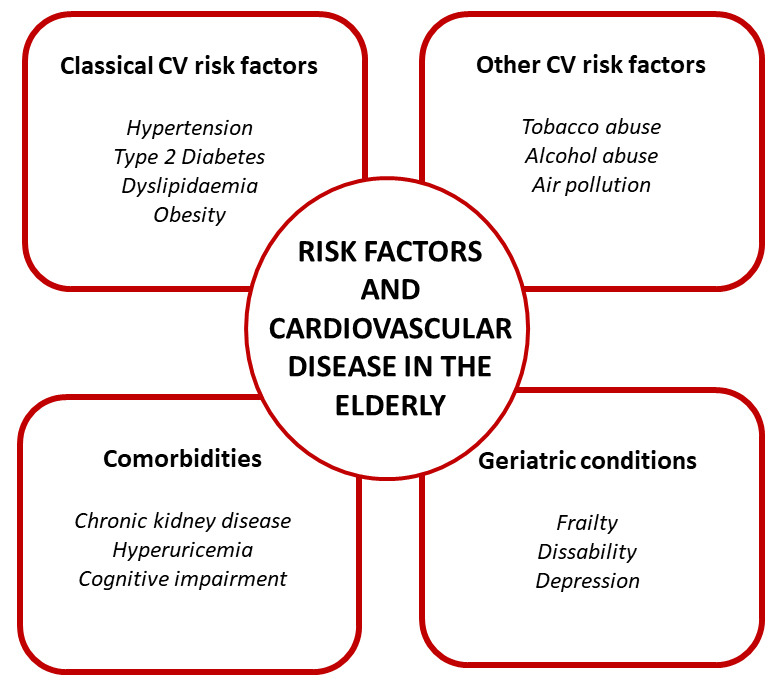
**Main cardiovascular risk factors involved in initiation and progression of cardiovascular disease in elderly population**. The most important cardiovascular risk factors are summarized, thus including classical risk factors, but also common in the elderly, such as certain comorbidities and geriatric conditions.

Age has been associated with increased cardiovascular (CV) risk factors and cardiovascular disease (CVD), being CV and cerebrovascular events the leading cause of morbidity and mortality in elderly population. However, not only age, but some other factors that play a key role in the development of CVD, ought to be known and addressed. Fig. 1 summarizes main CV risk factors involved in initiation and progression of CVD in elderly population, all of which are thoroughly reviewed in this text, also addressing the importance of primary and secondary interventions aimed to improve both quality of life and life expectancy.

Age itself is the main risk factor for vascular disease, involving macrovascular and microvascular impairment [1, 2, 3, 4]. Age-dependent arterial injury clinical manifestations typically occur after the fifth or sixth decade of life, although there is a high individual variability in vascular disease onset as ageing is a heterogeneous process [2]. In addition, future research must improve the information about aging biomarkers and tools to identify more accurate aging indicators to better understand the different velocities of ageing [2].

Interventions for achieving health vascular aging are behavioural and pharmacological [2, 3]. They are aimed to achieve normal blood pressure and to reduce arterial stiffness. Among the pharmacological interventions, some are clearly established, such as antihypertensive agents and statins [2, 3]. Table 1 (Ref. [3]) shows a summary of the main interventions for achieving health vascular aging.

**Table 1.** **Main interventions for achieving health vascular aging and avoiding frailty**.

Strategies		Evidence
Lifestyle strategies		
	Aerobic exercise	Conflicting evidence for vascular ageing. Important evidence to avoid frailty
	Weight loss and total energy intake	In overweight and obese adults, it reduces arterial stiffness and blood pressure
	Healthy dietary patterns: High consumption of fruit and vegetables and/or Mediterranean diet	Conflicting evidence of arterial stiffness reduction. Evidence in blood pressure reduction.
		Evidence to avoid frailty
	Sodium restriction	Evidence in reduction of arterial stiffness and blood pressure.
	Flavonoids (citrus fruits, seeds, olive oil, tea, red wine, legumes)	Evidence in reduction of arterial stiffness.
Pharmacological strategy		
	Antihypertensive treatment	Evidence in reduction of arterial stiffness and blood pressure.
	Statins	Evidence in reduction of arterial stiffness.

Adapted from Nowak KL *et al*. [3].

## 2. Traditional Cardiovascular Risk Factors 

### 2.1 Hypertension

The prevalence of hypertension (HT) increases with age, especially for isolated systolic HT [5]. In adults ≥70 years, the estimated prevalence for HT is 73.6% for men and 77.5% for women in those belonging to high-income countries, and in those coming from low- and middle-income countries the prevalence is 65.6% for men and 74.7% for women [6]. In such patients, systolic blood pressure (SBP) appears to be a better predictor of events than diastolic blood pressure (DBP), since increased pulse pressure has additional adverse prognostic significance [7, 8, 9, 10, 11, 12, 13].

For many years, advanced age has been a barrier to the treatment of HT because of concerns about potential poor tolerability, and even harmful effects of BP-lowering interventions in people in whom mechanisms preserving BP homeostasis and vital organ perfusion may be more frequently impaired [14]. However, current evidence shows that in old and very old patients, antihypertensive treatment substantially reduces CV morbidity and CV and all-cause mortality. Of note, data from the HYVET (*Hypertension in the Very Elderly*) trial, the SPRINT (*Systolic Blood Pressure Intervention*) Trial, and the STEP (*Strategy of Blood Pressure Intervention in the Elderly Hypertensive Patients*) trial could reflect the benefit of more intensive BP reduction in relatively healthy octogenarians, more than the effect on frail patients [15, 16, 17]. On the other hand, older patients are more likely to have comorbidities such as renal impairment, atherosclerotic vascular disease, and postural hypotension, which may be worsened by BP-lowering drugs. Polypharmacy may also interact with BP control treatment.

According to this approach, current recommendations, as summarized on the European Society of Hypertension (ESH) and European Society of Cardiology (ESC) guidelines, which consider older as those patients with age ≥65 years and the very old as those with age ≥80 years, recommend emphasizing on biological rather than chronological age, also taking into account other aspects such as frailty, independence, and the tolerability of treatment [5]. Target of SBP and DBP are 130–139 (if tolerated), and <80 mmHg, respectively. As BP-lowering drug treatment, a two-drug combination, in a single pill combination, is recommended, though in very old patients it may be appropriate to initiate treatment with monotherapy. Antihypertensive treatment may also be considered in frail older patients if tolerated [16]. Close monitoring of adverse effects, especially orthostatic hypotension, is recommended and withdrawal of BP-lowering drug treatment based on age is not recommended if treatment is well tolerated [5]. Unless required for concomitant diseases, loop diuretics and alpha-blockers should be avoided because of their association with injurious falls [18, 19]. Renal function should be frequently assessed to detect possible increases in serum creatinine and reductions in estimated glomerular filtration rate (eGFR) because of BP-related reductions in renal perfusion.

### 2.2 Type 2 Diabetes

High prevalence of type 2 Diabetes (T2D) is especially important in the elderly population: more than 25% of people over 65 years have T2D and 50% of older adults have prediabetes [20]. Moreover, elderly patients with T2D have higher rates of heart disease, cerebrovascular disease, and stroke, than those without DM [21].

Achieving adequate glycemic control in the elderly with T2D continues to be a challenge, especially due to the great clinical, cognitive, and functional heterogeneity. Balancing risks and benefits of glycemic control is mandatory to establish an individualized reasonable glycosylated hemoglobin (A1C) goal in elderly patients [22]. In addition, age, comorbidities, and life expectancy must be considered. Usually, A1C in older patients should be maintained below 8.0% to prevent both complications and mortality. However, some studies have shown a U-shaped relationship between A1C and mortality, highlighting that strict glycemic control increases the risk of mortality in diabetic older patients [23]. The American Diabetes Association proposes a practical approach with different A1C goals for the elderly. Those who are healthy, with intact cognitive and physical functions and with long life expectancy should have an A1C <7.0–7.5%; older patients with several coexisting chronic illnesses or mild-to-moderate cognitive impairment should have and intermediate A1C goal (<8.0%). Finally, in patients with multiple coexisting chronic illnesses, poor health or moderate-to-severe cognitive impairment an A1C goal should be avoided and glucose control decisions should be based on avoiding hypoglycaemia and symptomatic hyperglycaemia [22].

T2D treatment interventions in the elderly should aim to prolong life but also to improve quality of life. Considering the heterogeneity of T2D older patients, both individualizing and simplifying (as well as assessing for drug interactions) the treatment should be mandatory [24]. Most T2D treatment clinical trials do not include elderly patients, which makes it necessary to consider data from studies including younger patients to establish treatment recommendations for the elderly [25]. Thus, drug side effects could be underestimated [26, 27].

Current treatment guidelines generally recommend the use of monotherapy with metformin as initial treatment for the elderly [22]. It can be safely used in patients with estimated glomerular filtration rate ≥30 mL/min/1.73 $m^{2}$, and the risk of hypoglycemia is low [28]. When T2D older patients do not achieve their A1C target with metformin, it will be necessary to consider combination therapy with two or more antidiabetic drugs, or insulin. The selection of the appropriate drug should be based on efficacy in A1C reduction, relative risk of hypoglycemia, adverse effects, and CV profile [29].

Insulin is often used in elderly patients with A1C >9% or with persistent symptomatic hyperglycemia. Its use requires appropriate visual, motor, and cognitive skills. Once-daily basal insulin treatment is a reasonable option in many older patients [22]. Insulin dose will be lowest possible at baseline and should be titrated to meet individualized glycemic targets and to avoid hypoglycemia. Multiple-daily injections can be a challenging option for older people with cognitive/functional limitation and increases the risk of hypoglycemia [24].

Sulfonylureas and thiazolidinediones should be used with caution in elderly patients. Sulfonylureas are associated with high risk of hypoglycemia and thiazolidinediones with congestive heart failure (HF), osteoporosis, and bone fractures [22].

Regarding incretin-based therapies, studies performed with dipeptidyl-peptidase-4 (DPP-4) inhibitors in elderly patients confirmed the efficacy of these drugs, which are well tolerated, with few side effects and very low risk of hypoglycemia [30, 31, 32]. Despite this, saxagliptin should be used cautiously, if used at all, in older T2D patients because they may increase risk of hospitalization for HF, particularly in patients with history of previous HF or chronic renal disease, based on data from SAVOR TIMI 53 (*Saxagliptin and Cardiovascular Outcomes in Patients with Type 2 Diabetes Mellitus*) [33].

GLP1 receptor agonists main benefits are efficacy in A1C reduction and weight loss, cardiorenal protection, and negligible risk of hypoglycemia [34]. Despite this, GLP1 receptor agonists are injectable agents (except for oral semaglutide) so visual, motor, and cognitive abilities are required for appropriate administration [35]. Moreover, given weight loss and their gastrointestinal side-effects, these drugs should be avoided in frail patients, particularly those with malnutrition [29]. Cardiorenal benefits seem to be consistent also in the elderly. Post-hoc analysis of the LEADER (*Liraglutide Effect and Action in Diabetes: Evaluation of CV Outcome Results*) study, with 9% of the study population ≥75 years old, found elderly patients had a 34% risk reduction in the frequency of MACE and a 35% in all-cause mortality [35]. Post-hoc analysis of the SUSTAIN-6 (*Semaglutide and Cardiovascular Outcomes in Patients with Type 2 Diabetes*), with 43% of patients ≥65 years, showed once weekly semaglutide reduced the risk of the first occurrence of MACE and each MACE component consistently across all age subgroups, compared with placebo [36].

Last, but not least, sodium-glucose co-transporter 2 (SGLT2) inhibitors have demonstrated efficacy in A1C reduction, very low risk of hypoglycemia, and great CV benefits in patients with established atherosclerotic CVD or heart failure. Moreover, these agents slow the progression of chronic kidney disease [37]. This cardio-renal protective effect appears to emerge early. A systematic review and meta-analysis of SGLT2 inhibitors CV outcome trials showed that the protective effect was consistent across age categories, and elderly constituted about 50% of the total participants in the three major SGLT2 inhibitors trials [38, 39, 40]. SGLT2 inhibitors use is safe in elderly patients, although they should be used cautiously in patients with previous genitourinary infections, and in older patients with factors predisposing to diabetic ketoacidosis [29].

Cardio-renal protection results of SGLT2 inhibitors and GLP1 receptor agonists in elderly patients are impressive. Therefore, their use in older people is likely to increase. However, evidence for those above the age of 80 years or frail individuals with multiple comorbidities is still lacking.

### 2.3 Obesity

Obesity is associated with an increased risk of developing CVD. Despite this, in patients over age 75, relative risk of death from all causes and CVD has been found to decrease with increasing body mass index (BMI) [41]. Individuals with class I obesity present a more favorable prognosis compared to individuals who are normal or underweight. Thus, several studies have identified a BMI of 24 to 35 as “ideal”. This phenomenon is called the obesity paradox, and it is particularly evident in HF [42]. However, BMI could be an imperfect measure of obesity in the elderly, and most studies suggesting the existence of this puzzling paradox could have underestimated other key aspects as body composition, visceral adiposity, and sarcopenic obesity.

### 2.4 Dyslipidemia

Dyslipidemia is defined as elevated total or low-density lipoprotein (LDL) cholesterol levels above 90th percentile, or low levels of high-density lipoprotein (HDL) cholesterol below 10th percentile [43]. Ageing is associated with impaired lipid metabolic pathways, leading to higher LDL-cholesterol and triglycerides levels due to less degradation. Cholesterol levels progressively increases from puberty, reaching a plateau until 70 years and then those levels persist or fall slightly [44]. Not surprisingly, dyslipidemia is estimated to affect almost 40% of population over 65 years, with those with higher plasma LDL-cholesterol levels entailing the higher risk of atherosclerotic disease and acute cardiac events [45, 46].

Clinical guidelines support the same recommendations that in younger ones regarding secondary prevention. They recommend a goal-directed therapy, with a reduction ≥50% of LDL-cholesterol levels, reaching a target of LDL-cholesterol <55 mg/dL in very high CV risk and <70 mg/dL in high CV risk [47]. Accordingly, a recently published meta-analysis showed that lipid lowering therapies effectively reduce CV death, myocardial infarction, stroke and coronary revascularisation in patients aged ≥75 years [48].

Treatment involves lifestyle modification and pharmacologic treatment. Statins are the most prescribed drugs. They are considered safe in elderly patients, with some studies demonstrating clinical benefits also in primary prevention [49, 50]. Other pharmacologic tools include ezetimibe, PCSK9 inhibitors, bempedoic acid and inclisiran. Ezetimibe, used alone or combined with statins, associates CV benefits in elderly patients with a safe profile in specific clinical studies [51, 52]. Regarding evolocumab and alirocumab, sub-analysis from FOURIER (*Further Cardiovascular Outcomes Research With PCSK9 Inhibition in Subjects With Elevated Risk*) and Odyssey (*Evaluation of Cardiovascular Outcomes After an Acute Coronary Syndrome During Treatment With Alirocumab*) trials demonstrated CV benefits also in elderly patients [53, 54]. Inclisiran and bempedoic acid are considered the most recent treatments for dyslipidemia. Both drugs have showed to provide a safe and effective reduction in LDL levels in patients over 65 and 75 years of age, similar to their younger counterparts [55, 56].

Finally, yet importantly, we recommend dyslipidemia treatment to be adapted to baseline patient specific conditions such as frailty or polypharmacy, also considering the risk of drug interaction, thus minimizing possible side effects and improving compliance to treatment, which in turn associates greatest clinical benefits [57].

### 2.5 Other Cardiovascular Risk Factors: Air Pollution, Alcohol and Tobacco

Recent studies have demonstrated a most pronounced effect of air pollution on health status in older adults. Air pollution with fine particulate matter has been associated with frailty, particularly in the very elderly or in those with low incomes [58]. The underling mechanisms might be pollution-associated oxidative stress and inflammatory status. Furthermore, a recent critical review showed a consistent effect of air pollution on cognition impairment and dementia, probably due to neuro-inflammation and reduction in white matter volume [59]. Heart failure seems to be one of the cardiac conditions most influenced by air pollution, with different contaminants like ozone ($O_{3}$), nitrogen dioxide (${\mathrm{NO}}_{2}$) and sulfur dioxide (${\mathrm{SO}}_{2}$) being associated with heart failure hospitalizations in the elderly [60].

Chronic alcohol intake is associated in older adults with higher body mass index and blood pressure, as well as atherosclerotic events [61, 62]. Paradoxically, alcohol consumption and risk of incident frailty are inversely related. In a recent meta-analysis the highest alcohol consumption was associated with lower frailty risk (odds ratio = 0.61, 95% confidence interval = 0.44–0.77) although two of the four individual studies suggested a U-shape association with lowest risks for moderate drinkers [63]. This might be explained by a poorer basal heath status in nondrinkers; especially cognition, depressive symptoms, education, comorbidities, and self-reported general health. The main limitations of these studies were that alcohol consumption was mainly self-reported and that consumption pattern was completely different among countries. In fact, a Mediterranean drinking pattern, defined as moderate alcohol intake, with wine preference and drinking only with meals, has been associated with a lower risk of frailty [64]. One of the possible explanations to this finding could be that alcohol is often consumed socially; so moderate consumption frequently means an active social life.

Tobacco consumption has a direct effect in the cardiac structure and function [65]. Besides, compared with non-smokers, smokers are more likely to develop frailty, which seems to be related with the increase in mental and physical illnesses directly associated to smoking [66].

## 3. Other Conditions and Comorbidities Associated with Cardiovascular Disease in the Elderly

Several comorbidities may impact on the development of CVD or interfere with CV risk factors. The prevalence of all these factors increases with age, as well as the comorbidity burden, thus they often coexist. Comorbidities must be considered when assessing CV risk in the elderly, because they increase the risk of non-CV mortality and traditional risk scores may overestimate CV risk [67]. Decreased life expectancy and quality of life may modify the aims of the CV prevention, especially in patients with severe or multiple comorbidities [68]. Nevertheless, we must not fall into therapeutic nihilism and abandon the control of CV risk factors in the elderly patient. Therefore, a comprehensive approach to the patient and his comorbidities is key, and goals must be individualized. It is advisable that multidisciplinary teams and close collaboration between primary care and specialists lead to a holistic patient management, in which all their circumstances are considered when making decisions [67].

Chronic kidney disease is highly prevalent in the older patient, and share pathophysiological features with CVD [69]. Aging itself is associated with changes in renal anatomy and physiology which led to a reduction of glomerular filtration, but this renal function decline is multifactorial and CV risk factors play an important role [69]. On the other hand, severe impairment of renal function is considered as a major CV risk factor, and intensive control of classical risk factors should be aimed [67]. However, several challenges are encountered when dealing with CV risk control in this setting, including difficulties in dose adjustment, interactions, and lack of specific evidence. Statins must be included for lipid control, but high-dose regimes should be avoided [70]. Recent evidence supports the use of PCSK9 inhibitors in mild to moderate renal dysfunction [71]. Specific benefits may be expected with the use of angiotensin-converting enzyme (ACE) inhibitors or angiotensin receptor blockers (ARB) for HT and SGLT2 inhibitors for T2D, as previously addressed, due to their renoprotective effects [72].

There is evidence supporting the relationship between serum uric acid and CV risk factors in the older patient, since elderly individuals with hyperuricemia have a higher prevalence of obesity, HT, lipid profile alterations and impaired glucose metabolism [73]. There is also a close relationship between dietary intake of purine-rich food (mainly meat) and high uric acid levels, while vegetables consumption has a protective effect [74]. A subanalysis of the PREDIMED (*PREvención con DIeta MEDiterránea*) trial including 4449 elderly patients at high CV risk, found that the adherence to a mediterranean diet is associated with a lower risk of hyperuricemia [75].

Cognitive impairment is closely related to CVD. CV risk factors and other pathophysiological pathways are common in both entities [76]. Moreover, they show a two-way relationship: cognitive impairment may hinder therapeutic control and adherence, and the development of CV events is associated with a progressive deterioration of mental status [77]. There is an obvious relationship between CV risk factors and cerebrovascular disease, as it can be considered as a different clinical manifestation of the same disease. Interestingly, in the older patient, evidence of cerebrovascular lesions in the absence of any stroke history is a common finding at neuroimaging, and it is associated to a higher prevalence of CV risk factors and marker of increased risk of stroke [78]. Additionally, depression is common in the older patient, which may overlap with cognitive decline and impact on CV outcomes. Therefore, special attention should be made on cognitive function and depression screening in these patients.

## 4. Role of Frailty and Other Geriatric Conditions 

Frailty is a clinical state with increased vulnerability to stressors, due to a decline in physiological reserve and function. It is usually related to age, but different to disabilities. Frailty increases CV morbidity and mortality and has been associated to conservative management and poorer clinical outcomes [79, 80]. Several tools for frailty assessment have been developed, and some questions regarding the moment for frailty measurement, which tool to use in each clinical setting and which clinical decisions must be taken remain open. In summary, there are two approaches to assess frailty, using a physical phenotype or using a multidomain approach. The frailty physical phenotype is a clinical syndrome with three or more of the following criteria: unintentional weight loss, self-reported exhaustion, weakness, slow gait speed, and low physical activity. In this approach frailty is a predictor of disability, being this latter the result of frailty. On the contrary, multidomain approach considers frailty as the result of deficits in multiple domains, being disability one of them. Usually, in patients older than 65 years old, we assess disability using a Barthel scale and, when it is not present, we assess physical frailty. When disability is present, we use a comprehensive geriatric assessment [80].

When deciding the best prevention strategy in older patients we should consider both disabilities and frailty. Firstly, a comprehensive geriatric assessment should be performed. If the patient has physical or cognitive disability, high burden comorbidity or reduced life expectancy, CV risk quantification with CV risk scales is useless. If not, physical frailty should be assessed. If the patient is robust, conventional CV risk scales would be used for primary prevention, as if the patient were younger than 75 years old. If the patient is frail or prefrail, clinicians should consider the potential reversibility of frailty, and consider changes in diet and physical activity to do so. Clinicians should carefully individualize decisions in these patients. In addition, if a patient is frail, CV disease should be excluded. If present, the patients should be treated according to current clinical practice and secondary prevention could be appropriate [81]. Currently, new options as the frailty team and eHealth are going to better manage frailty [79].

Optimal control of CV risk factors is essential not only to prevent CVD, CV mortality and hospital admission but also to mitigate the economic burden of CVD. The economic impact of CVD is increasing worldwide, as a consequence of both progressive ageing of population and increasing prevalence of CV risk factors. HF, which represents the leading cause of hospitalization in patients over 65 years old, represents an estimated expense of more than 100 billion dollars annually. Thus, achieving a good control of predisposing factors such as HT or T2D is fundamental [82].

## 5. Other Risk Factors for the Promotion of Cardiovascular Conditions in the Elderly (Fig. 2)

### 5.1 Oxidative Stress

There is increasing evidence that age itself is associated with an imbalance between reactive oxygen and nitrogen species production and neutralization by endogenous antioxidants. This disparity may partially explain the age-related functional decline [83]. Oxidative stress in the elderly has been related with increased levels of oxidized LDL-cholesterol (oxLDL). OxLDL easily accumulates in the arterial wall, promoting atherosclerosis, independently of the other CV risk factors [84]. Besides of that, oxidative stress contributes to vascular endothelial dysfunction and vascular remodelling, leading to HT and atherosclerosis disease. Healthy diet and physical activity reduce oxidative stress particles; however, more evidence is needed and target treatments should be developed [83].

**Fig. 2.**
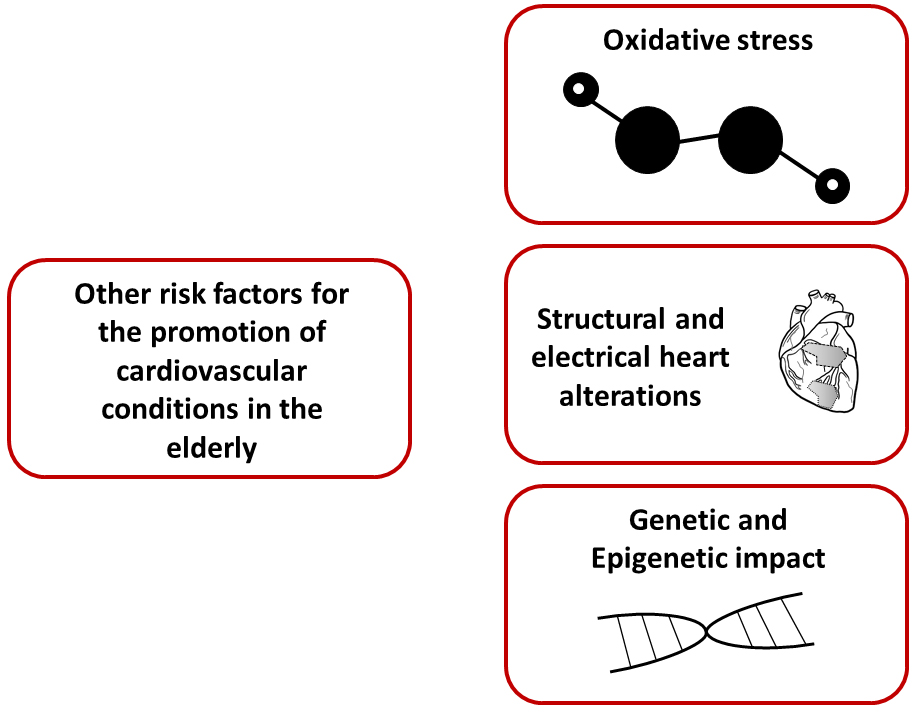
**Other risk factors for the promotion of cardiovascular conditions in the elderly**. Oxidative stress, structural and electrical heart alterations, together with genetic and epigenetic factors, may be involved in the development of cardiovascular disease in the elderly.

### 5.2 Structural and Electrical Heart Alterations 

Ageing is associated with structural changes in the heart such as development of myocardial fibrosis, decline in diastolic function and left atrial dilatation. Conduction system is also affected, with decrease in intrinsic heart rate and conduction delays [85]. Both, structural and electrical changes are associated with an increase in the prevalence of atrial fibrillation (AF) in this population. AF itself appears to be associated with increased cardiovascular risk, especially in women [86]. Older patients with AF have higher rates of stroke, bleeding and death [87]. It has been suggested that early intervention and control of CV risk factors reduces AF burden and may improve maintenance of sinus rhythm [67]. Optimal anti-coagulation therapies, especially with direct oral anticoagulants (DOAC), should be provided in order to reduce adverse outcomes. Direct oral anticoagulants use was superior to warfarin in reducing stroke or systemic embolization in elderly AF patients without a significant increase in bleeding risk [88].

### 5.3 Genetic and Epigenetic Impact

Genetics represents a revolution in cardiovascular field. It is already known its importance in the aetiology of many cardiovascular diseases, but the genetic information is not currently used in cardiovascular risk factors scores. Additional evidence is still required; however, family history of CVD should be compiled [67]. Moreover, recent evidence has shown that lifestyle and environmental factors may affect genetic expression. Genetic information may be significantly altered by different mechanisms globally known as epigenetics. Those mechanisms include DNA methylation, histone acetylation, and miRNA expression. All of them are mainly induced by environmental factors (such as pollution, smoking) or chronic inflammation. Not only they may lead to premature atherosclerosis and CVD but also they may be transmitted to the offspring [89].

## 6. Secondary Prevention and Cardiac Rehabilitation

Secondary prevention objectives in older adults do not differ much to their younger counterparts, as shown in Table 2, but particular attention on drug side effects, overdosing and intolerance is recommended [90]. The cornerstone of the treatment is acquiring a healthy lifestyle, based on dietary habits, regular exercise and tobacco cessation. The accomplishment of these three goals can lead to optimal weight, better control of the CV risk factors, and reduction of morbimortality [91].

**Table 2.** **Secondary prevention objectives**.

Cardiovascular risk factor	Recommendation
Tobacco	Complete cessation
Weight	Body mass index 20–24.9 kg/$m^{2}$
Diet	Mediterranean pattern
Exercise	Aerobic (5 days per week)
	Resistance (2 days per week)
	Flexibility/Balance (5 days per week)
Hypertension	<140/90 mmHg
LDL-cholesterol	<55 mg/dL (<1.4 mmol/L)
Diabetes: A1C levels	<7–7.5% in fit healthy individuals
	<8% in frail individuals
Cardiac Rehabilitation Program	Referral if possible

Dietary counselling is one of the bases of the control of the risk factors, but malnutrition is more prevalent in older people and it should be addressed, even in patients with normal weight [92]. A diet based on fruits, vegetables, legumes, and fish and polyunsaturated fats, like the Mediterranean pattern, is advised [93]. Regular exercise should be divided in moderate intensity aerobic sessions (at least 30 minutes, 5 days per week), moderate resistance training (2 non-consecutive days per week), flexibility and balance exercises (10 minutes, 5 days per week). Exercise can be adapted to osteomuscular disabilities or individual preferences of the patients [94].

Tobacco cessation is mandatory, as benefits of its withdrawal are observed independently to age [95]. Nicotine replacement therapy have been proved safe and effective in older adults. Bupropion and varenicline can be useful, but evidence is weaker and there is concern of their neuropsychiatric effects in the elderly [96].

Blood pressure target is less than 140/90 mmHg. The stricter goal of less than 130/80 mmHg, when tolerated, has also shown benefits in healthy older adults, but it remains unknown its effects on the very frail, where drug-to-drug interactions, orthostatic hypotension and subsequent risk of fall, are more frequent. Closer control of adverse effects and slow titration of medication is recommended. In secondary prevention, ACE inhibitors and ARBs are preferred over other choices [97].

LDL cholesterol goal is less than 55 mg/dL (<1.4 mmol/L). High potency statins are the cornerstone of the pharmacological treatment, and there is no evidence of more muscle-related symptoms than in younger patients. However, polypharmacy has to be addressed again, as the probability of interactions rise [57]. As previously explained, ezetimibe and PCSK9 inhibitors seem to be also effective and safe [53, 54].

Regarding diabetes, goals depend on life expectancy and risk of adverse events. In healthy older adults, a level of A1C <7–7.5% (53–58 mmol/mol) can be achieved, especially under treatments with low risk of hypoglycaemia and disease self-management. When those conditions are lacking, a more lenient goal of A1C <8% (64 mmol/mol) should be of choice to avoid either hypoglycaemia or acute hyperglycaemic states. In type 1 diabetes, continuous glucose monitoring is useful in reducing hypoglycaemia episodes. In type 2 diabetes, overweight and obesity are usually related conditions, and those patients should be encouraged to losing weight, given its benefits, with SGLT2 inhibitors and GLP-1 receptor agonists prescription depending on clinical situation. The use of insulin, sulfonylureas and meglitinides has to be extremely careful because of the risk of hypoglycaemia and should be reduced or even withdrawn when diet and exercise adherence improve or when new diabetes drugs are prescribed [22, 98].

Cardiac Rehabilitation Programmes (CRPs) have shown morbimortality benefits in older patients [99]. CRPs can handle the peculiarities of the secondary prevention in the elderly. However, older patients are referred less often than younger ones. Nevertheless, given the progressive aging of the population, one third of the patients attending CRPs are over 75 years old. This is why frailty should be routinely addressed in CRPs, although the scale to do so is yet to be determined [100]. Scales including physical performance as modified Fried scale or the SPPB test could be of use [80].

The exercise program must be individualized, with longer periods of warming and cooling. Sudden movements should be avoided. Both central and peripheral functional limitations are improved, and so is pain control [101]. Moderate-vigorous aerobic and resistance exercise has beneficial CV and non-CV effects in the elderly. In this sense, tailored rehabilitation programs are especially appropriated to older population, since CRP present a similar benefit in older people after a CV event than in younger patients [102]. Exercise training is, associated to an increase in exercise duration, peak oxygen consumption, and ventilatory threshold in older patients with chronic heart failure with reduced ejection fraction [103]. Nevertheless, whether exercise training can reduce mortality, hospitalizations, and overall health care costs in older adults with CVD is still under research [104].

The effects of exercise in non-CV outcomes in elderly people have also been analyzed. The evidence is controversial regarding the incidence of falls with some data supporting a protector effect of exercise in pre-frail but not in frail patients and other suggesting no effect of training on the number of falls [105, 106]. Functional improvement and increased muscle strength after training programs also support the beneficial effect of exercise [107]. Although rarer in younger patients, nutritional deficits, cognitive decline and social/familiar support should be routinely taken into account in elder patients, as these comorbidities can alter the usual approach to control de classic CV risk factors. Exercise, combined with nutrition supplementation might even reverse frailty and prevent cognitive impairment [108, 109]. In a recent multicenter clinical trial, a transitional progressive rehabilitation intervention showed a greater improvement in physical function than usual care [110]. Finally, higher fitness level identifies older people with good long-term survival regardless CV risk factors burden [111].

## 7. Conclusions

CV risk factors are highly prevalent in the elderly. Not only traditional CV risk factors, such as HT, T2D, dislipemia or obesity, but also other CV risk factors such as tobacco or alcohol abuse and air pollution significantly impact in the long-term prognosis. Additionally, specific comorbidities as chronic kidney disease, hyperuricemia, or cognitive impairment should be taken into account. Although genetics, ageing-related structural and electrical heart changes and oxidative stress play a key role in CV disease in the elderly, additional studies addressing these issues are required. Also geriatric syndromes such as frailty, are highly prevalent in the elderly and closely related with CVD. Hence, primary and secondary interventions are of great importance since they reduce both morbidity and mortality in the elderly. Of note, such interventions should consider baseline conditions, including life expectancy and quality of life, thus providing most adequate care to patients’ necessities.
